# An Integrative Model of Tourists’ Pro-Environmental Behavior Based on the Dual Path of Rational Planning and Embodied Emotion

**DOI:** 10.3390/ijerph19137910

**Published:** 2022-06-28

**Authors:** Xingping Cao, Junlin Qiu, Leyu Wang, Gefen Zhou

**Affiliations:** Business and Tourism School, Sichuan Agricultural University, Dujiangyan 611830, China; cxp@sicau.edu.cn (X.C.); qiujunlin97@outlook.com (J.Q.); leyu0525@outlook.com (L.W.)

**Keywords:** embodied theory, theory of planned behavior, engagement with nature, connectedness to nature, pro-environmental behavioral intentions

## Abstract

Tourists’ pro-environmental behavior is one of the key factors for the sustainable development of natural scenic spots. Although this behavior depends on the surroundings and context, the existing literature lacks the perspective of specific scenarios, especially that of embodied emotions. This research integrated the theory of planned behavior and embodied theory to construct an integrative model of pro-environmental behavior that combined tourists’ “rational planning” and “embodied emotion” and conducted an empirical study. The results show that in natural scenic spots, “rational planning” and “embodied emotion” affect tourists’ pro-environmental behavior simultaneously on dual paths, and factors such as behavioral attitude, perceived behavioral control, subjective norm, engagement with nature, and connectedness to nature have different effects on high- and low-effort pro-environmental behavioral intentions. The findings of the study provide a new explanatory perspective for individual pro-environmental behaviors and a basis for effectively predicting and guiding tourists’ pro-environmental behaviors in natural scenic spots.

## 1. Introduction

The original beauty of the natural ecosystem is the core attraction of natural scenic spots. The environmental misbehavior of tourists undermines the attractiveness of scenic spots, reduces the quality of tourists’ experience and hinders the sustainable development of scenic spots [1,2]. Pro-environmental behavior refers to behavior that benefits the environment or harms the environment as little as possible [3]. Other concepts are similar, such as green behavior, environmentally responsible/supportive behavior, ecological behavior, and environmentally friendly behavior [4]. It is necessary to guide tourists’ pro-environmental behaviors and reduce environmental anomie behaviors, such as dropping litter carelessly [4,5], trampling on grass [6], and destroying ecology [7], to maintain the stability of ecological structures and the sustainable development of vulnerable, natural scenic spots. Guiding tourists to actively engage in pro-environmental behaviors will effectively reduce management costs, improve tourist experiences, and promote the sustainability of the tourism industry [8].

The current literature is mainly based on theories of rational behavior to explain the formation mechanism of pro-environmental behaviors and its intentions, such as the theory of planned behavior (TPB), the norm-activation model (NAM), and value-belief-norm theory (VBN). The trend of “reason dominates” is ongoing [9,10]. As the research continues to deepen, some empirical studies on the relationship between rational cognition factors and pro-environmental behavior have deemed it nonsignificant [11]; however, it must be wondered whether another interpretation exists of individuals’ pro-environmental behaviors in addition to demographic, sociocultural, and rational cognitive factors. Environmental psychologists have found positive correlations between emotion factors and pro-environmental behaviors [12,13], and in some situations, emotion factors have a stronger explanatory power than cognition factors [14]. Ives et al. (2018) believe that strengthening emotional connectedness to nature is more effective for addressing ecological and sustainability challenges [15].

Individuals often obtain information from their surroundings to decide whether to adopt pro-environmental behaviors; therefore, situational factors are considered to be important variables affecting pro-environmental behaviors [10,16] such as formal regulations [17] and policies [18]. Based on that, management agencies frequently used to regulate and guide tourists’ environmental behaviors by formulating environmental policies, corresponding regulations, or standards of conduct in practice. However, when formulating environmental strategies, scenic spots lack necessary consideration for the stimulation and guidance of tourists’ environmental emotions in specific scenes.

Connectedness to nature (NC) is defined as the degree of people’s emotional connection and belonging to nature [19], which can positively promote environmental values, attitudes, and behavior [20,21]. With the development of environmental education practices advocating “deeply integrating into nature and experiencing nature”, researchers began to pay attention to the positive effect of exposure to nature on environmental emotion, environmental attitude, values, and behavioral change [22,23,24]. Enactive emotion is usually triggered by situational or environmental elements; for example, guiding children to contact nature can strengthen their NC [25,26].

While some tourists actively engage in pro-environmental behaviors, most tourists’ travel motivations are almost for hedonic purposes, without proactively considering environmental conditions [27]. Compared with mass tourists, ecotourists have more positive, pro-environmental behavioral intentions [28,29]. For many mass tourists with stable environmental attitudes and values, it is wondered whether we can enhance their NC by guiding deep interaction and physical engagement in the natural environment to promote their pro-environmental behaviors. Empirical studies are needed to verify the effectiveness of this mechanism.

Therefore, this study integrates TPB and embodied theory and explores the influence mechanism of pro-environmental behavioral intention from the dual paths of rational planning and embodied emotion, specifically to further verify the influence of three key variables of behavioral attitude, subjective norm, and perceived behavioral control in TPB on pro-environmental behavior and the mediating role of connectedness to nature in the relationship between engagement with nature and pro-environmental behaviors.

## 2. Literature Review, Hypothesis, and Research Model

### 2.1. The Rational Interpretive Framework and Influencing Factors of Tourists’ Pro-Environmental Behaviors in Natural Scenic Spots

The TPB, based on social psychology, notes that people’s behavioral intentions directly determine their behavior, and behavioral intentions are determined by three factors: behavioral attitude, subjective norm, and perceived behavioral control [30]. The TPB provides a basic theoretical framework with high explanatory power and is the most used for explaining individual environmental behaviors. The TPB has also shown good predictive power for pro-environmental behaviors in different contexts, such as low-carbon travel tool selection in green hotels [31], recycling behavior in the workplace [32], waste sorting [33], energy saving and recycling environmental behavior in daily life [34,35], and green consumption behavior [36]. Based on the TPB, researchers have continuously incorporated other variables and proposed the norm-activation model (NAM) and value–belief–norm theory (VBN) to explain individual pro-environmental behavior.

TPB, NAM, and VBN all prove the important role of rational driving factors in individual pro-environmental behaviors. The NAM studies individual prosocial behaviors based on norms and social bonds [37]. Research indicates that the perception of consequences affects the attribution of responsibility and drives the moral obligation to inspire prosocial behaviors [3], such as organic vegetable buying [38], willingness to pay for environmental protection [39], and resource recycling [40]. VBN theory further integrates worldviews, values theory, and new environmental paradigm theory into the NAM interpretation framework [41]. The impacts of an ecological worldview and biosphere values are further discussed in this framework to explain how environmental behavior attitude forms [4]. In sum, three TPB factors are significant variables: behavioral attitude, subjective norm, and perceived behavioral control.

Pro-environmental behavior is a highly contextualized concept that is not consistent across different situations [42]. Specifically, people’s pro-environmental behavioral intentions differ from one situation to another [43,44,45]; therefore, studies of pro-environmental behaviors must consider different social and geographical contexts [46].

Contextual differences between natural environments and social relationships shape different person–spatial and interpersonal interactions that in turn facilitate or inhibit pro-environmental behaviors [47]. Tourism is an activity of escaping from one’s habitual residence [48]; thus, in it, the impact of environmental situational factors on individual behavior is further strengthened [45,49,50,51].

In traveling, people are in anonymous identity states and with hedonic motivations; as a result, they tend to take less pro-environmental behaviors when away from their daily environments [52]. On the one hand, tourists tend to weaken the moral norm requirements for their behaviors due to public pressure [53]; on the other hand, the time, energy, and other resource inputs for tourists’ pro-environmental behaviors may conflict with their hedonic motives. Meanwhile, the unfamiliarity of the destination or the scenic environment also reinforces such resource input expectations, further increasing tourists’ psychological costs of resource input. Therefore, when people are removed from their familiar surroundings and enter a tourism context, their pro-environmental behaviors may tend to follow emotional rather than rational principles [54]. Therefore, behavioral attitude, subjective norm, and perceived behavioral control in the TPB model have been considered to explain how rational factors affect the pro-environmental behaviors of tourists in natural scenic spots.

Behavioral attitude refers to the degree to which an individual evaluates the performance of a particular behavior favorably or unfavorably [30], and here it refers specifically to tourists’ favorable or unfavorable evaluations of their own pro-environmental behavior. According to TPB, behavioral attitude is an important variable influencing behavioral intentions: The more positive an individual’s attitude toward a behavior, the stronger their behavioral intentions. Conversely, the more negative an individual’s attitude toward a behavior, the weaker their behavioral intentions [55]. Behavioral attitude is effectively predicted in pro-environmental behavioral willingness, such as purchasing sustainable products [56]. The literature has shown that tourists’ attitudes toward environmentally responsible behavior affect their behavioral willingness [57]; thus, we propose H1:

**Hypothesis** **1** **(H1).** *Behavioral attitude has a significant positive effect on tourists’ pro-environmental behavioral intentions.*

Perceived behavioral control refers to the perceived ease or difficulty of performing a particular behavior [35]. When people have more resources (e.g., time or money) and more required skills and abilities, perceived behavioral control is greater, and high levels of perceptual behavioral control result in stronger behavioral intentions [30,58,59]. Perceived behavioral control is suggested as a critical factor in individuals’ pro-environmental behavioral intentions [58,60,61], such as recycling behavior online and off-line [62,63], daily green purchasing behavior [64,65], community forestry behavior [66] and litter management behavior [67,68]. Thus, we hypothesize that:

**Hypothesis** **2** **(H2).** *Perceived behavioral control has a significant positive effect on tourists’ pro-environmental behavioral intentions.*

Subjective norm refers to the perceived social pressure to perform or not perform a behavior [30]. People’s socially oriented group expectations, as a form of social pressure, will have an impact on individuals’ abilities to adopt specific behaviors or make specific decisions [69]. Azjen (1980) suggested that consumers’ behavioral intentions are influenced by their perceived social pressure [70]. The relevant literature verified the significant influence of subjective norm on environmental behavior [71,72] and pro-environmental behavioral intentions [66,73,74,75,76]; hence, we hypothesize that:

**Hypothesis** **3** **(H3).** *Subjective norm has a significant positive effect on tourists’ pro-environmental behavioral intentions.*

### 2.2. The Affective Interpretive Framework of Tourists’ Pro-Environmental Behaviors in Natural Scenic Spots

Morrison and Robinson (1997) found that emotions can elicit attention to specific pro-environmental behaviors [77] and that emotional factors have significantly higher explanatory power than rational factors in green consumption contexts [14]. However, emotional factors have not received sufficient attention in long-term studies [78]. With the deepening understand of pro-environmental behavior, emotional factors have been gradually regarded as an important breakthrough in recent studies [79].

An increasing number of studies have shifted focus from rational cognitive factors to emotional factors elicited in different contexts, providing an emotion-driven explanatory pathway for pro-environmental behavioral research. Research has confirmed that anger, empathy, guilt, place attachment, and other emotional factors all had significant effects on individual pro-environmental behaviors [80,81,82,83,84]. With the development of urbanization, people hope to regain their emotional connections to nature, and the relationship between people and nature is deeply considered in research as emotional factors such as awe of nature [85], NC [20,86], and natural empathy [81,87] receive more attention. Especially in the post-COVID pandemic era, a growing number of scholars have been showing interest in environmental emotional factors [88,89].

Context plays an important role in arousing emotions [90]. In recent years, scholars have begun to increasingly emphasize the enactive orientation of emotions [91], arguing that emotional experiences are embodied and embedded contextually and that emotions are triggered by different contexts, arising from the interactions and couplings of the brain, body, and concrete environment [92]. In addition to the polarity of emotions (i.e., positive and negative) [93,94,95,96], specific emotions induced by different contexts and aroused by natural tourism scenarios, such as a sense of awe [97], empathy [81,98], or guilt [99], also have a significant impact on pro-environmental behavioral intentions [100].

Embodied theory is concerned with the roles of situational individual actions and bodily states in human psychology and behavior [101], and it emphasizes the embodied and interplay relationships of bodies and the environment [102,103,104]. The traditional mind–body dualism assumes that human mental processes can exist independent of the body; conversely, embodied theory focuses on the dependence of the mind on the body and emphasizes the oneness of mind and body, and it is believed that embodied scenarios such as natural exposure can have a significant impact on changing human cognition, emotion, and behavior [105].

People capture information about the environment using their senses, which are considered to be the basis of personal interaction with the surroundings [106]. With the promotion of embodied theory, researchers have focused on the “scene absence” [107] and “body absence” [108] in the study of tourist behavior and advocated returning to tourist subjectivity and embodied experiences though their sensory engagement [109]. Increasingly, studies are focusing on the interactions between tourists’ bodies, perceptions, and external environments in specific times and spaces [110,111].

Tourists acquire perceptions and experiences of their surroundings in tourism scenarios resulting in direct emotional connections to the tourism destinations, which influence their pro-environmental behavioral intentions [112,113]. It has been suggested that the embodied experience of a heterogeneous context and environmental perceptions has a significant effect on the willingness to engage in pro-environmental behaviors [113,114].

Mass tourists often use multiple senses including sight, hearing, smell, taste, and touch to perceive the external environment [115], which generates a variety of emotions [116]. When tourists are embedded in a natural context, varying degrees of natural emotions and pro-environmental behavioral intentions may be inspired through multisensory channels [117]. Research has shown that the more holistic a tourist’s perception of the environment is, the more pro-environmental behavior occurs [118]. However, most of the literature focusing on embodied experience is about one or a few specific senses [119,120], and it lacks consideration of the integrity of physical experience [121].

Engagement with nature refers to individuals’ interactions with the natural environment while performing physical activity [122]. In the perspective of embodied experience, unlike the concept of nature contact, engagement with nature emphasizes the “mutual embeddedness” of the individual body and the surroundings; it has the specific connotation of the subject’s actively integrating into the surroundings behaviorally and the body’s proactive and conscious sensory touch and active construction of the surroundings. Individual cognition, perception, and differences in external stimuli during tourism activities can lead to different levels of physical engagement with the natural environment [123,124]. The human senses are interactively integrated [125], and experiences generated by different senses can enrich a subject’s other sensory experiential processes to optimize the overall experience; meanwhile, tangible multisensory information can enhance positive experiences [126] and lead to more immersion in the environment [124].

In natural scenic spots, tourists and the surroundings form an inter-embedded organism. In objective environmental contexts, with individual personality differences, tourists interact with the surroundings through their senses to form different experiences and perceptions using all five senses [127], such as visual experience [128], sound landscape perception [129], and olfactory landscape perception [124], resulting in corresponding environmental psychology, emotions, and behaviors. Studies show that tourist perceptions and engagement significantly improve their environmental protection behaviors in ecotourism contexts [123], and children’s free exploration in the woods is more likely to promote pro-environmental behavior compared with tree planting tasks [130]. Active forms of engagement in nature can promote a deeper appreciation of nature and the subsequent practice of sustainable behaviors [131,132]. We thus propose H4:

**Hypothesis** **4** **(H4).** *Engagement with nature has a significant positive impact on tourists’ pro-environmental behavioral intentions.*

Many studies have shown that the senses have the function of emotional arousal, which is a direct physiological effect. For example, smells are an important trigger for nostalgic emotions in tourism, with local smells often evoking memories and closeness [133]. Warm colors cause higher levels of anxiety than cool colors, while cool colors initiate emotions such as calmness and love [134]. Tourist experiences are multisensory [120], and traveling is more likely to trigger positive emotions than daily life due to the rich sensory stimuli in tourism situations [117]. NC is the degree to which people are emotionally connected to and belong to nature [19]. This positive emotional connection to nature has important implications for individual well-being [22]. Research has shown that even short-term experiences with nature can stimulate participants’ NC [135] and that increased exposure to nature can advance this state of connectedness [23]. Different levels of engagement with nature can trigger different sensory experiences, sensory imagery [136], and psychological states [122]. The more frequent contact with nature and the deeper one’s experiences, the more positive one’s emotional attitude toward nature and the higher their degree of NC [137,138]. Based on this, we propose H5:

**Hypothesis** **5** **(H5).** *Engagement with nature has a significant positive effect on connectedness to nature.*

Emotions are complex physical and mental states with initiative power that influence individuals’ responses to the environment [139]. Positive emotions toward nature can promote pro-environmental behavior [140,141], and it is only when people experience themselves as part of the natural world at the emotional level that they become more empathetic, concerned about the state of nature, and willing to engage in environmental conservation actions. It has been shown that enhancing individuals’ NC in a certain place can help promote their pro-environmental behaviors [20,142,143,144,145]. Otto and Pensini (2017) demonstrated that children’s NC can better predict their pro-environmental behavior with an explanation rate of over 60% [137]; therefore, we propose H6:

**Hypothesis** **6** **(H6).** *Connectedness to nature has a significant positive effect on tourists’ pro-environmental behavioral intentions.*

### 2.3. Research Model

**Figure 1:**
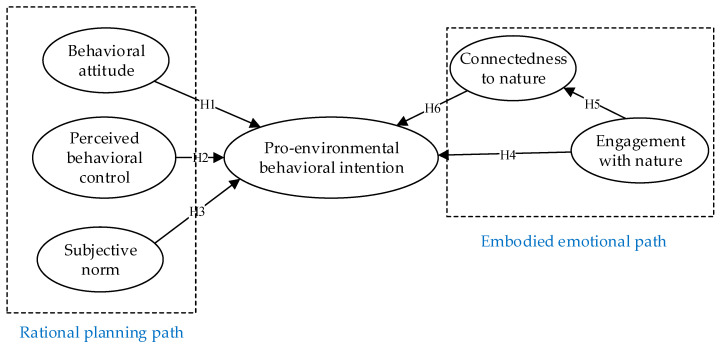
The study research model.

This study combined the TPB with embodied theory and constructed an integrated model of the relationships between behavioral attitude, perceived behavioral control, subjective norm, engagement with nature, connectedness to nature, and pro-environmental behavioral intentions. The research model is shown in Figure 1.

## 3. Methodology

### 3.1. Measures

Engagement with nature (NE), which was referenced from Han and Wang (2018), included a total of five observed variables to measure the extent to which tourists interact with nature through sight, hearing, smell, taste, and touch [122]. Connectedness to nature (NC) was referenced from Richardson et al. (2019), with six measurement items revised according to context [146]. The items for behavioral attitudes (BA), perceived behavioral control (PC), and subjective norm (SN) were referenced from Song et al. (2012) [147]. We structured pro-environmental behavioral intentions by referring to Kerstetter et al. (2004) [148] and Ramkissoon et al. (2013) [149], and seven items were retained according to the research context (see Appendix A).

Pro-environmental behaviors are influenced by environmental objectivity and individual subjective initiative. In tourism scenarios, individuals are bound by mandatory rule requirements and thus passively choose to perform compliance-based pro-environmental behaviors. However, some individuals may also actively choose to perform pro-environmental behaviors with higher difficulty and level of engagement [44,150] and higher resource investment [46] and to practice pro-environmental behaviors in various consumption parts of tourism [151,152,153]. Thus, tourists’ pro-environmental behavior decisions include whether to adopt pro-environmental behaviors and how many resources and efforts they are willing to commit to. The extensive literature often treats tourists’ pro-environmental behavior as a unidimensional variable, ignoring its diversity and multidimensionality. However, a growing number of researchers have begun to focus on different dimensions of tourists’ pro-environmental behaviors and distinguish them into low-effort and high-effort behaviors [45,149], which is valuable for exploring the influence factors and psychological mechanisms of active pro-environmental behaviors.

Then, two variables of high-effort pro-environmental behavioral intention (HPEBI, including four items) and low-effort pro-environmental behavioral intention (LPEBI, including three items) were constructed based on different levels of invested resources (time, money, and energy) when implementing pro-environmental behavior actions (see Appendix A). The Cronbach’s α of the two variables are greater than 0.7, indicating that each variable has the desired internal consistency [154]; thus, H1–H4 and H6 were divided into two hypotheses.

**Hypothesis** **1a** **(H1a).** *Behavioral attitude has a significant positive effect on tourists’ high-effort pro-environmental behavioral intentions.*

**Hypothesis** **1b** **(H1b).** *Behavioral attitude has a significant positive effect on tourists’ low-effort pro-environmental behavioral intentions.*

**Hypothesis** **2a** **(H2a).** *Perceived behavioral control has a significant positive effect on tourists’ high-effort pro-environmental behavioral intentions.*

**Hypothesis** **2b** **(H2b).** *Perceived behavioral control has a significant positive effect on tourists’ low-effort pro-environmental behavioral intentions.*

**Hypothesis** **3a** **(H3a).** *Subjective norm has a significant positive effect on tourists’ high-effort pro-environmental behavioral intentions.*

**Hypothesis** **3b** **(H3b).** *Subjective norm has a significant positive effect on tourists’ low-effort pro-environmental behavioral intentions.*

**Hypothesis** **4a** **(H4a).** *Engagement with nature has a significant positive impact on tourists’ high-effort pro-environmental behavioral intentions.*

**Hypothesis** **4b** **(H4b).** *Engagement with nature has a significant positive impact on tourists’ low-effort pro-environmental behavioral intentions.*

**Hypothesis** **6a** **(H6a).** *Connectedness to nature has a significant positive effect on tourists’ high-effort pro-environmental behavioral intentions.*

**Hypothesis** **6b** **(H6b).** *Connectedness to nature has a significant positive effect on tourists’ low-effort pro-environmental behavioral intentions.*

The final questionnaire measured the respondents’ demographic characteristics—including gender, age, and education, in addition to the core variables—on a 7-point Likert scale.

### 3.2. Data Collection and Participants

The research team randomly intercepted tourists for questionnaire surveys at different locations in the posterior part of Mount Qingcheng on October 6–7 and 29–30, 2021. Mount Qingcheng belongs to the World Heritage-Mixed Property Mount Qingcheng and Dujiangyan irrigation system and is also one of the important parts of the World Natural Heritage Sichuan Giant Panda Sanctuary. With its rich biodiversity and Taoist cultural resources, Mount Qingcheng can be divided into two parts: the anterior part and the posterior part, with the anterior part having more cultural landscapes and the posterior part being covered by luxuriant and verdant trees and having evergreen scenery. A total of 490 questionnaires were obtained. After excluding invalid samples, a total of 416 usable responses were used for the hypothesis testing, with an effective rate of 84.90%. The sample profile is shown in Table 1.

## 4. Results

### 4.1. Measurement Model Test

First, the study tested the reliability and validity of the reflective variables. Factor loadings, composite reliability (CR), Cronbach’s α, and average variance extracted (AVE) were calculated, and the results are shown in Table 2.

The CR of each variable was greater than 0.8; Cronbach’s α were greater than 0.7; and AVEs were greater than 0.5, which satisfied the requirement of convergent validity for the latent variables. The discriminant validity of each latent variable is shown in Table 3.

For the formative variable, first, multicollinearity and weight significance were tested by referring to Cenfetelli and Bassellier (2009) [155]. The results showed that the variance inflation factor (VIF) of each NE item was less than 3.3, indicating no serious collinearity problem [156]. Then, the outer weights and outer loadings of the indicators were estimated using bias-corrected bootstrapping with 5000 replicate samples. The results (in Table 4) showed that all indicators had relatively high weights (outer weigher > 0.2, *p* < 0.05) except NE5. Specifically, the weights of NE5 were below 0.2 and nonsignificant (*p* = 0.124), but the loadings of NE5 were significant (*p* < 0.001). According to Cenfetelli and Bassellier’s (2009) suggestion, it is not comprehensive to decide whether to keep the items of formative latent variables only based on the significance of the weights, and these should be removed only if neither the indicator weights nor the loadings are significant [155]; therefore, NE5 was retained.

### 4.2. Structural Model Test

**Figure 2:**
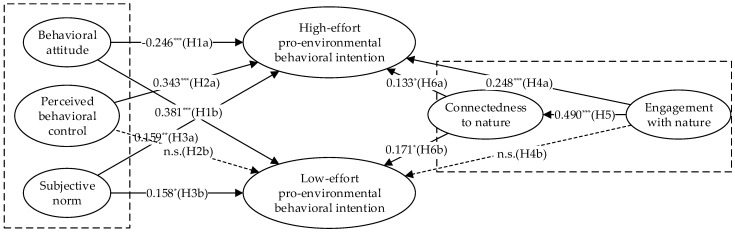
The structural equation model test results. Note: * *p* < 0.05; ** *p* < 0.01; *** *p* < 0.001; n.s. is nonsignificant.

We tested the hypotheses using the bootstrapping method with a sampling of 5000 times. As shown in Table 5, except for H1a, H2b, and H4b, the hypotheses were verified. The estimation results are shown in Figure 2.

To compare the explanatory power of the models, R-squared values of the TPB-based rational planning model (Model1), the embodied emotion model based on embodied theory (Model2), and the integrative model (Model3) were calculated separately. The results showed that the R-squared values for HPEBI and LPEBI were 0.236 and 0.319 in Model1, 0.227 and 0.144 in Model2, and 0.322 and 0.335 in Model3. Thus, the dual-path integrative model of “rational planning” and “embodied emotion” was proved to have stronger explanatory power for tourists’ pro-environmental behavioral intentions, as shown in Table 6.

### 4.3. The Mediation Analysis

To further verify the mediation effect of NC between NE and tourists’ pro-environmental behavioral intentions, bootstrapping (5000 times) was used. As shown in Table 7, the total, direct, and indirect effects of NE on HPEBI are significant, while the total and direct effects of NE on LPEBI are nonsignificant, and NC completely mediates both.

## 5. Discussion and Conclusions

### 5.1. Discussion

First, estimates in the rational planning pathway “behavioral attitude, subjective norm, perceived behavioral control → pro-environmental behavioral intention” revealed that BAs have a positively significant effect on low-effort pro-environmental behavioral intentions and a negatively significant effect on high-effort pro-environmental behavioral intentions. H1b was supported, but H1a was not. Then, it was demonstrated that PC has a significant positive effect on high-effort pro-environmental behavioral intentions; that is, H2a was supported, but the effect of PC on low-effort pro-environmental behavioral intentions (H2b) was not supported. The effect of SN on tourists’ pro-environmental behavioral intentions was positively significant for both high and low effort, meaning that H3a and H3b were supported. Studies in the ecological field showed that the link between attitudes and behavioral intentions is becoming increasingly complex [157]. Some studies have endorsed the significant positive effect of BAs on general pro-environment behavioral intentions [55,57]; however, in tourism contexts, Wang et al. (2020) found that the effect of cognitive attitudes on pro-environmental behavioral intentions was superficial or even negative [158]. In recent years, a number of scholars have also confirmed the relatively indirect effect of rational BAs on pro-environmental behavioral intentions [47] as well as the instable effect on different dimensions of pro-environmental behavior [143]. PC can promote high-effort pro-environmental behavioral intentions, which is consistent with most studies [4,159,160], and it has no significant impact on low-effort pro-environmental behavioral intentions, which is consistent with Zhang et al. (2018) [47]. A possible reason is that individuals do not need to consider excessive resource input when performing low-effort pro-environmental behaviors and if the behavior can be controlled by themselves [157]. When faced with the choice of high-effort pro-environmental behaviors, individuals must invest more effort, and PC over the environment can influence the degree of individual resource investment. The outcome of SN to PEBI is consistent with the majority of studies examining tourists’ pro-environmental behaviors [160,161,162,163,164]; that is, the stronger the subjective norms of tourists, the more they tend to produce varying degrees of pro-environmental behavioral intentions.

Second, the results of the embodied emotional pathway “engagement with nature → connectedness to nature → pro-environmental behavioral intention”, showed that engagement with nature has different effects on pro-environmental behavioral intentions with different levels of effort. NE has a significant direct effect on high-effort pro-environmental behavioral intentions, and H4a was supported; NC played a partially mediating role. However, NE had a nonsignificant direct effect on low-effort pro-environmental behavior intentions, H4b was rejected, and NC played a fully mediating role between the two factors. The results further show that tourists’ embodied engagement with nature positively affected their NC, which means that enhancing tourists’ experiences of their five senses in nature can significantly increase their emotional connectedness to nature. H5 was supported. Additionally, NC has significant direct effects on high- and low-effort pro-environmental behavioral intentions; that is, H6a and H6b were supported. In the embodied emotional path, the finding is consistent with the conclusions of Dopko et al. (2019) and Matteucci (2016) that the more the senses are evoked, the more positive experiential emotions are triggered [135,165]. By promoting a deeper appreciation of nature [132], the emotional connection to nature can be enhanced [138]. NC has been used as a relatively constant affective variable in many studies [23], and some studies have also indicated that tourists’ NC can be evoked in natural contexts [166]. The findings here further demonstrate the methodological significance of guiding tourists’ environmental behaviors by creating a deep, five-sense experience to evoke emotion toward nature.

### 5.2. Conclusions

The conclusions confirm the different impacts of three rational factors on individual pro-environmental behavior in natural scenic spot tourism contexts from that in other situations. From an embodied experience perspective, this study proved that active engagement with nature in natural contexts can directly and indirectly enhance tourists’ intentions to engage in pro-environmental behaviors, especially high-effort pro-environmental behaviors. Meanwhile, it is valuable to explore different types of pro-environmental behaviors, and the different effects of rational attitudes on the influence of high- and low-effort pro-environmental behavioral intentions in the tourism context deserve to be further discussed.

## 6. Theoretical and Practical Implications

### 6.1. Theoretical Implications

This study combined the TPB and embodied theory to verify the dual internal drives of embodied emotion and rational planning in pro-environmental behavior. The existing literature has explored the factors influencing pro-environmental behavior and its psychological mechanisms mostly from a rational perspective. Although some studies have verified the profound influence of emotional factors, especially NC, on pro-environmental behavior, they have not further explored the antecedents of NC, and nor have they tested the two parallel paths in an integrative model.

In this study, the role of bodily participatory practices that integrate tourists’ senses into nature in shaping NC and pro-environmental behaviors is explored in depth. Although embodied philosophy has been used as a guideline for practice in some fields, the research on how embodied experiences change individuals’ emotions toward NC and pro-environmental behaviors is still lacking. The existing literature has demonstrated that exposure to nature can promote individuals’ perceptions of nature and the self and contribute to the generation of positive emotion [166]. However, the research has mainly regarded the environment as an object opposite to individuals [167,168], and it has not examined the individual as embedded in the surroundings; thus, the current research lacks a perspective related to individual–environment interactions. Therefore, breaking through the guest (resource) perspective of individual exposure to nature, based on embodied theory, this study introduces the core concept of NE, highlighting the significance of tourists’ subjective bodily practices.

### 6.2. Managerial Implications

The ecological environment is the foundation of the sustainable development of natural scenic spots, but current norm-based practices have not paid enough attention to tourists’ subjectivity in improving their environmental misbehavior and guiding their pro-environmental behavior. According to the results from this study, scenic spots can attempt to guide tourists’ pro-environmental behaviors through both a rational planning path and an embodied emotion path to promote sustainable environmental development.

Firstly, based on the rational path, to guide tourists’ pro-environmental behaviors, except for forming social norms, improving the convenience of environmental facilities and interpretation systems is still a useful measure for enhancing tourists’ efficacy and reducing the threshold of tourists’ perceived behavior control. Secondly, the scenic spots should create more natural atmosphere, allowing tourists to engage with the natural surroundings and guiding them to open their sensory channels, which can not only improve tourists’ NC and provide a more positive emotional experience but also guide more sustainable tourist behavior.

## 7. Limitations and Future Research

Differences in scenic environmental elements as situational factors trigger different level of NE, NC, and pro-environmental behaviors among tourists. Due to the COVID-19 epidemic, this study only chose Mount Qingcheng as a research site; although the research team selected different places on the mountain and different attractions to reinforce environmental differences, our efforts objectively weakened the influence of environmental factors on tourists’ embodied perceptions and psychological and environmental behaviors. Instead, we focused more on tourists’ own perceptual acuity and differences in perceived outcomes. In addition, although this study’s survey sample involved all age groups, more than half of the respondents were younger, and the sample distribution was uneven; therefore, future researchers should focus on the generalizability of the research findings. Future studies should further incorporate objective contextual factors such as landscape quality, degree of crowding, and facilities and equipment as important control variables for consideration.

## Figures and Tables

**Table 1:** Sample profile.

Profiles		Number	Percentage (%)
Gender	Man	210	50.50
	Female	206	49.50
Age	≤18	29	6.97
	19–28	223	53.61
	29–38	118	28.37
	39–48	29	6.97
	49–58	14	3.37
	≥59	3	0.72
Education	Middle school or below Middle school	21	5.05
	High School/Vocational School	43	10.34
	College	81	19.47
	University	214	51.44
	Master	53	12.74
	Ph.D.	4	0.96

**Table 2:** The measurement model test results for the reflective variables.

Reflective Variables	Items	Factor Loadings	CR	Cronbach’s α	AVE
Behavioral attitude	BA1	0.838	0.899	0.831	0.748
	BA2	0.842			
	BA3	0.914			
Perceived behavioral control	PC1	0.761	0.844	0.722	0.643
	PC2	0.799			
	PC3	0.844			
Subjective norm	SN1	0.864	0.885	0.804	0.720
	SN2	0.794			
	SN3	0.885			
Connectedness to nature	NC1	0.624	0.876	0.831	0.543
	NC2	0.698			
	NC3	0.794			
	NC4	0.761			
	NC5	0.760			
	NC6	0.771			
High-effort pro-environmental behavioral intention	HPBI1	0.841	0.893	0.840	0.676
	HPBI2	0.794			
	HPBI3	0.863			
	HPBI4	0.812			
Low-effort pro-environmental behavioral intention	LPBI1	0.776	0.848	0.734	0.651
	LPBI2	0.848			
	LPBI3	0.770			

Note: CR = Composite reliability; AVE = Average variance extracted.

**Table 3:** The latent variable discriminant validity results.

	NE	BA	PC	SN	NC	HPEBI	LPEBI
NE	Formative construct						
BA	0.194	0.865					
PC	0.314	0.337	0.802				
SN	0.264	0.664	0.410	0.849			
NC	0.490	0.404	0.409	0.414	0.737		
HPEBI	0.415	0.076	0.458	0.256	0.361	0.907	
LPEBI	0.175	0.549	0.250	0.473	0.377	0.155	0.898

Note: The diagonal elements are the square roots of all constructs’ AVEs. Unadjusted correlations among constructs are below the diagonal. Engagement with nature (NE); Behavioral attitude (BA); Perceived behavioral control (PC); Subjective norm (SN); Connectedness to nature (NC); High-effort pro-environmental behavioral intention (HPEBI); Low-effort pro-environmental behavioral intention (LPEBI).

**Table 4:** Assessing the engagement with nature measurement model.

Formative Variables	Items	VIF	Outer Weights		Outer Loadings	
			Estimate	*p* Value	Estimate	*p* Value
Engagement with nature	NE1	1.305	0.351	0.002	0.696	0.000
	NE2	1.450	0.260	0.003	0.694	0.000
	NE3	1.608	0.249	0.027	0.752	0.000
	NE4	1.403	0.402	0.000	0.738	0.000
	NE5	1.367	0.148	0.124	0.617	0.000

Note: Variance inflation factor (VIF).

**Table 5:** The results of the path coefficient test.

Hypothesis	Path Coefficient	T Value	*p* Value	Support
H1a: BA → HPEBI	−0.246	4.771	0.000	No
H1b: BA → LPEBI	0.381	5.045	0.000	Yes
H2a: PC → HPEBI	0.343	6.279	0.000	Yes
H2b: PC → LPEBI	−0.007	0.136	0.891	No
H3a: SN → HPEBI	0.159	2.630	0.009	Yes
H3b: SN → LPEBI	0.158	2.419	0.016	Yes
H4a: NE → HPEBI	0.248	3.475	0.001	Yes
H4b: NE → LPEBI	−0.022	0.369	0.714	No
H5: NE → NC	0.490	11.117	0.000	Yes
H6a: NC → HPEBI	0.133	2.443	0.015	Yes
H6b: NC → LPEBI	0.171	2.418	0.015	Yes

Note: Behavioral attitude (BA); Perceived behavioral control (PC); Subjective norm (SN); Engagement with nature (NE); Connectedness to nature (NC); High-effort pro-environmental behavioral intention (HPEBI); Low-effort pro-environmental behavioral intention (LPEBI).

**Table 6:** R-squared values for the individual and integrative models.

Explained Variables	Rational Planning Model (Model1)	Embodied Emotion Model (Model2)	Integrative Model (Model3)
HPEBI	0.236	0.227	0.322
LPEBI	0.319	0.144	0.335

Note: High-effort pro-environmental behavioral intention (HPEBI); Low-effort pro-environmental behavioral intention (LPEBI).

**Table 7:** The mediation effects of NC.

Explained Variable	Effect	Estimate	SD	*p* Value	95% Confidence Interval
High-effort pro-environmental behavioral intention	Total effect	0.313	0.061	0.000	(0.188, 0.428)
	Direct effect	0.248	0.070	0.000	(0.102, 0.377)
	Indirect effect (Via NC)	0.065	0.028	0.020	(0.013, 0.122)
Low-effort pro-environmental behavioral intention	Total effect	0.062	0.050	0.203	(−0.038, 0.158)
	Direct effect	−0.022	0.059	0.710	(−0.134, 0.096)
	Indirect effect (Via NC)	0.084	0.034	0.018	(0.017, 0.151)

Note: Standard deviation (SD); Connectedness to nature (NC).

## Appendix A

**Table A1:** Measuring the Constructs.

Construct	Items
Engagement with nature	NE1 Actively observe the plants or animals in the scenic spot.
	NE2 Actively listen to the natural sounds in the scenic spot.
	NE3 Actively feel the natural smell from the surroundings.
	NE4 Try the flavors of some plant in the scenic spot if it is safe and licensed.
	NE5 Actively touch the plants or animals in the scenic spot if it is safe and licensed.
Behavioral attitude	BA1 Protecting the environment of scenic spots is a valuable behavior.
	BA2 Protecting the environment of scenic spots is a necessary behavior.
	BA3 Protecting the environment of scenic spots is a beneficial behavior.
Perceived behavioral control	PC1 It is entirely up to me to protect the environment of scenic spots.
	PC2 I can protect the environment of scenic spots.
	PC3 I am confident that I can protect the environment of scenic spots if I want.
Subjective norm	SN1 Most people who are important to me support that I protect the environment of scenic spots.
	SN2 Most people who are important to me understand that I protect the environment of scenic spots.
	SN3 Most people who are important to me agree with me that I protect the environment of scenic spots.
Connectedness to nature	NC1 I feel the beauty in nature.
	NC2 I treat nature with respect.
	NC3 Being in nature makes me very happy.
	NC4 Spending time in nature is very important to me.
	NC5 I find being in nature amazing.
	NC6 I feel myself part of nature.
High-effort pro-environmental behavioral intention	HPEBI1 In scenic spots, I will proactively pick up the garbage thrown by others.
	HPEBI2 In scenic spots, I will express my opinion to the local administration if I find the phenomenon of environmental pollution or destruction.
	HPEBI3 In scenic spots, I will provide some volunteer work for environmental protection.
	HPEBI4 In scenic spots, I will voluntarily donate money for environmental needs.
Low-effort pro-environmental behavioral intention	LPEBI1 In scenic spots, I will properly deal with my garbage.
	LPEBI2 In scenic spots, I will follow the environmental policy.
	LPEBI3 In scenic spots, I will encourage friends not to litter.
